# Wild-type *Lactococcus lactis* producing bacteriocin-like prophage lysins

**DOI:** 10.3389/fmicb.2023.1219723

**Published:** 2023-07-14

**Authors:** Timo M. Takala, Samira Mokhtari, Susanna L. Ahonen, Xing Wan, Per E. J. Saris

**Affiliations:** ^1^Department of Microbiology, Faculty of Agriculture and Forestry, University of Helsinki, Helsinki, Finland; ^2^Expert Microbiology Unit, Finnish Institute for Health and Welfare, Helsinki, Finland

**Keywords:** *Lactococcus*, prophage, endolysin, virion-associated lysin, bacteriocin

## Abstract

**Introduction:**

*Lactococcus* is a genus of lactic acid bacteria used in the dairy industry as a starter. Lactococci have been found to produce altogether more than 40 different bacteriocins, ribosomally synthesized antimicrobial proteins. All known *Lactococcus* spp. bacteriocins belong to classes I and II, which are mainly heat-resistant peptides. No class III bacteriocins, bigger heat-sensitive proteins, including phage tail-like bacteriocins, have been found from the *Lactococcus* spp. Unlike phage tail-like bacteriocins, prophage lysins have not been regarded as bacteriocins, possibly because phage lysins contribute to autolysis, degrading the host's own cell wall.

**Methods:**

Wild-type *Lactococcus lactis* strain LAC460, isolated from spontaneously fermented idli batter, was examined for its antimicrobial activity. We sequenced the genome, searched phage lysins from the culture supernatant, and created knock-out mutants to find out the source of the antimicrobial activity.

**Results and discussion:**

The strain LAC460 was shown to kill other *Lactococcus* strains with protease- and heat-sensitive lytic activity. Three phage lysins were identified in the culture supernatant. The genes encoding the three lysins were localized in different prophage regions in the chromosome. By knock-out mutants, two of the lysins, namely LysL and LysP, were demonstrated to be responsible for the antimicrobial activity. The strain LAC460 was found to be resistant to the lytic action of its own culture supernatant, and as a consequence, the phage lysins could behave like bacteriocins targeting and killing other closely related bacteria. Hence, similar to phage tail-like bacteriocins, phage lysin-like bacteriocins could be regarded as a novel type of class III bacteriocins.

## 1. Introduction

*Lactococcus lactis* is a lactic acid bacterium used as a starter culture for the acidification of many fermented dairy products (Cavanagh et al., 2015). In addition to the defined starters used in the modern dairy industry, *L. lactis* is frequently present in spontaneously fermented milk or plant-based foods. As an example, after spontaneous overnight fermentation, the ground rice–black gram batter for making the soft Indian breakfast cake idli contains many different species and strains of lactic acid bacteria, often including *Lactococcus* (Iyer et al., 2013). Many strains of *Lactococcus* produce bacteriocins, ribosomally synthesized proteins, which kill other bacteria (Acedo et al., 2018). Bacteriocins have various potential applications in the pharmaceutical industry as antibacterial agents, as well as in the food industry as preservatives (Soltani et al., 2021).

Based on their structure and biochemical properties, bacteriocins of Gram-positive bacteria are usually classified into three major groups (Acedo et al., 2018), even though other kinds of classifications have also been proposed (Soltani et al., 2021). Class I includes post-translationally modified, small (< 10 kDa) heat-stable peptides, and class II includes non-modified, small (< 10 kDa), mainly heat-stable peptides. The controversial group of class III bacteriocins is bigger (>10 kDa) heat-labile proteins, which can further be divided into subgroups (Acedo et al., 2018). Bacteriolysins are lytic enzymes that degrade bacterial cell walls. Well-characterized examples of this subclass are enterolysin A and lysostaphin from *Enterococcus* and *Staphylococcus*, respectively (Schindler and Schuhardt, 1964; Nilsen et al., 2003). Another subgroup of class III bacteriocins is non-lytic large bacteriocins, for example, dysgalacticin from *Streptococcus dysgalactiae* (Swe et al., 2009). In addition, a special type of class III bacteriocins is phage tail-like bacteriocins (PTLB), also known as tailocins, which are very large (>1,000 kDa) protein structures resembling the tail of bacteriophages, and function by penetrating the bacterial envelope causing the loss of membrane potential (Scholl, 2017). Phage tail-like bacteriocins have been found from many bacterial species, such as diffocins from *Clostridioides difficile*, and monocins from *Listeria monocytogenes* (Gebhart et al., 2012; Lee, 2020). In addition to PTLB, prophages may carry other genes and biosynthetic gene clusters encoding antibacterial proteins, which can be beneficial for the host (Dragoš et al., 2021). For example, colicin-like bacteriocins klebicins from *Klebsiella*, and the S-linked glycocin sublancin 168 from *Bacillus subtilis* are encoded by prophages (Chavan et al., 2005; Oman et al., 2011). According to the online bacteriocin databases Bactibase, Bagel4, and Labiocin, more than 40 bacteriocins have been found from *Lactococcus* species (http://bactibase.hammamilab.org/main.php; http://bagel4.molgenrug.nl/; https://labiocin.univ-lille.fr/). All known *Lactococcus* spp. bacteriocins are peptides belonging to classes I and II. Neither larger bacteriocins of class III, nor phage-associated bacteriocins have been identified.

Bacteriophage lysins are peptidoglycan (PG) hydrolases, which degrade the PG layer of the host bacteria. Phages often employ two different lysins at different stages of their life cycle: a virion-associated lysin (VAL) to locally degrade the bacterial peptidoglycan at the initial step of infection, and an endolysin breaking the cell wall at the end of the phage lytic cycle releasing the newborn phages from the host cell (Fernandes and São-José, 2018; Abdelrahman et al., 2021). Typically, endolysins of phages targeting Gram-positive bacteria have a modular structure. They are composed of two main domains: an enzymatically active domain (EAD) that hydrolyzes PG, and a cell-wall binding domain (CBD) that confers the ability to recognize substrates and binding (Abdelrahman et al., 2021). Owing to the diversity of their EADs and CBDs, endolysins exhibit very high specificity. Endolysins are usually co-produced with holin proteins, which form pores in the cell membrane, and thus mediate the release of endolysins allowing them to reach the cell wall (Abdelrahman et al., 2021). Therefore, endolysin and holin genes are typically located close—but not necessarily adjacent—to each other in phage genomes (Canchaya et al., 2003; Goh et al., 2007; Dorscht et al., 2009). VALs are structural components of the phage particle, often (but not always) attached to the phage tail, giving them the commonly used name tail-associated lysin (Latka et al., 2017). Similar to endolysins, VALs have a modular structure. However, even though VALs may carry one or two EADs, they often lack CBD, as the recognition and binding of the phage onto the cell are based on the interaction of other proteins on the phage particle (Oliveira et al., 2018). As an exceptional example, a cell binding region has been recognized from the VAL of *S. aureus* phage P68 (Takác and Bläsi, 2005).

Most, if not all, bacteria carry intact or defect prophages in their chromosomes, and, consequently, also genes encoding phage lysins. These lysin genes may be expressed in bacteria, with or without other phage activity. In such a case, the secreted lysins contribute to the degradation of the host cell wall, thus functioning like autolysins (Visweswaran et al., 2017). As prophage lysins target the cell wall of their host, they have not been considered as bacteriocins in wild-type bacteria. However, in genetically engineered recombinant strains producing heterologous endolysins or VALs from phages of other bacterial species, phage lysins can show high antimicrobial activity not affecting the host but acting like highly specific narrow-range bacteriocins (Gaeng et al., 2000; Grabowski et al., 2021; Chandran et al., 2022).

In this study, we report the identification of three secreted prophage-encoded lytic enzymes from a wild-type *L. lactis* strain. None of the three lysins lysed the host strain, but two of them showed bacteriocin-like activity against other lactococci.

## 2. Materials and methods

### 2.1. Bacterial strains and growth conditions

*Escherichia coli* and *Lactococcus* strains used in this study are listed in Table 1. *E. coli* strains were grown in LB at 37°C with 180 rpm shaking, and *Lactococcus* strains were grown in M17 (Oxoid, Basingstoke, UK) supplemented with 0.5% (w/v) glucose (M17G) at 30°C. *Bacillus, Enterococcus, Listeria, Micrococcus, Staphylococcus*, and *Streptococcus* strains were cultured in BHI (Lab M, Lancashire, UK), lactobacilli and *Weissella* strains in MRS (Oxoid), and *Leuconostoc* strains in calcium-citrate media (Wan et al., 2015) supplemented with 1% glucose, lactose, or sucrose. Growth conditions of these bacterial strains used as indicators in antimicrobial range tests are listed in Supplementary Table 1. For transformant selection and plasmid maintenance, erythromycin (Erm) was used at final concentrations of 250 μg/ml for *E. coli* and 10 μg/ml for *L. lactis*.

**Table 1:** Strains and plasmids used in this study.

**Strain or plasmid**	**Relevant properties**	**Reference/source**
**Strains**		
*Escherichia coli* TG1	Cloning host	Sambrook et al., 2001
*E. coli* ECO851	TG1 carrying *lysL* deletion vector pLEB815	This study
*E. coli* ECO852	TG1 carrying *lysT* deletion vector pLEB817	This study
*E. coli* ECO853	TG1 carrying *lysP* inactivation vector pLEB819	This study
*Lactococcus cremoris* MG1614	Indicator strain; Sensitive to LysL, resistant to LysP	Gasson, 1983
*Lactococcus lactis* LAC460 = HAMBI 3742	Wild-type strain isolated from fermented idli batter; producer of LysL, LysP, LysT	This study; HAMBI culture collection, University of Helsinki, Finland
*L. lactis* LAC468	LAC460(*lysP72*::pLEB819); *lysP* knock-out strain carrying thermosensitive plasmid pLEB819 inserted in the *lysP* gene, Erm^R^, 37°C	This study
*L. lactis* LAC470	LAC460(ΔL); *lysL* knock-out strain	This study
*L. lactis* LAC472	LAC460(ΔT); *lysT* knock-out strain	This study
*L. lactis* LAC526	LAC460(ΔTL); *lysL* and *lysT* double knock-out strain	This study
*L. lactis* LAC527	LAC460(ΔT, *lysP72*::pLEB819); *lysP* and *lysT* double knock-out strain, Erm^R^, 37°C	This study
*L. lactis* LAC528	LAC460(ΔL, *lysP72*::pLEB819); *lysL* and *lysP* double knock-out strain, Erm^R^, 37°C	This study
*L. lactis* LAC529	LAC460(ΔLT, *lysP72*::pLEB819); *lysL, lysP, lysT* triple knock-out strain, Erm^R^, 37°C	This study
*L. lactis* LM0230	Indicator strain; Sensitive to LysP, resistant to LysL	Efstathiou and McKay, 1977
*L. lactis* N8	Control strain, resistant to LAC460 CFS	Valio Ltd, Helsinki, Finland; Wan et al., 2021
**Plasmids**		
pG^+^Host4	Thermosensitive (TS) *Lactococcus* cloning vector containing TS*repA* and *ermC*	Maguin et al., 1992
pLEB815	LysL deletion plasmid; TS*repA* and *ermC* from pG^+^Host4, and two ~1-kb fragments from LAC460 chromosome for deleting *lysL* gene	This study
pLEB817	LysT deletion plasmid; TS*repA* and *ermC* from pG^+^Host4, and two ~1-kb fragments from LAC460 chromosome for deleting *lysT* gene	This study
pLEB819	LysP inactivation plasmid; TS*repA* and *ermC* from pG^+^Host4, and a 347-bp fragment for integration into *lysP* gene in LAC460 chromosome	This study

### 2.2. Characterization of antimicrobial activity and preparation of CFS concentrates

Antimicrobial activity was determined by the conventional spot-on-lawn method, as described previously (Wan et al., 2015). Cell-free supernatants (CFS) were prepared from overnight cultures by pelleting cells by centrifuging 7,000 *g* for 10 min and filtering the supernatant (pH 5.3) with a sterile 0.22 μm syringe filter. Overnight cultures or filtered CFS were spotted (e.g., 10 μl) on indicator bacteria lawn on suitable agar media, and the plates were incubated at the optimal temperature of the indicators (Supplementary Table 1).

To determine the proteinaceous nature of the antimicrobial substance, the LAC460 CFS was treated with different proteases, including pronase E (Merck KGaA, Darmstadt, Germany), proteinase K (Merck), and trypsin (Sigma-Aldrich, St. Louis, MO, USA). The proteases were added to CFS in 1 mg/ml final concentrations, and incubated for 2 h at 37°C. Then, the mixtures were spotted on the *Lactococcus cremoris* MG1614 indicator lawn, and the antimicrobial activity was assessed the next day as a formation of an inhibition zone. To determine the effect of temperature on the antimicrobial activity, 300 μl of filtered CFS was kept at −20, 4, 37, 45, 53, 60, or 75°C for 15 and 60 min, after which 10 μl was spotted on *L. cremoris* MG1614 indicator lawn, and the antimicrobial activity was assessed next day as a formation of an inhibition zone. To determine the pH tolerance of the antimicrobial substance, the pH of the CFS was adjusted with HCl to pH 5, 4, 3, and 2, and with NaOH to pH 6, 7, 8, 9, 10, 11, and 12. The samples were kept at 4°C for 2 h, after which 10 μl was spotted on *L. cremoris* MG1614 indicator lawn, and the antimicrobial activity was assessed the next day with the formation of an inhibition zone.

For testing the antimicrobial activity of lysin knock-out strains, as well as for the cell leakage test and zymographic analyses, concentrated CFS samples were prepared. First, CFSs from 40 ml overnight cultures of the wild-type LAC460 and the knock-out strains Δ*lysL*, Δ*lysP*, Δ*lysT*, Δ*lysLP*, Δ*lysLT*, Δ*lysPT*, and Δ*lysLPT* were concentrated with Amicon centrifugal filter units of 30 kDa cut-off membrane (Merck). In order to remove erythromycin present in *lysP* knock-out cultures, as well as other small molecule impurities, the concentrates were washed twice with 10 ml PBS, followed by 30 kDa filterings by centrifuging. The final volume of the concentrates was approximately 2 ml, representing approximately 20-fold the concentration of the supernatant proteins.

### 2.3. Genome sequencing and sequence analyses

For the whole genome sequence, we extracted genomic DNA from a 2 ml overnight culture of *L. lactis* LAC460 using a MagAttract HMW DNA kit (Qiagen, Hilden, Germany) according to the supplier's protocol. The genome was sequenced by DNA Sequencing Service at the Institute of Biotechnology, University of Helsinki, Finland. DNA Template Prep Kit 2.0 (Pacific Biosciences, Menlo Park, CA, USA) was used to generate 3–10 kb gDNA fragments, and DNA/Polymerase Binding Kit P6 (Pacific Biosciences) to generate a DNA polymerase/template library complex. Reads were *de novo* assembled using the RS hierarchical genome assembly process (HGAP) version 3.0 implemented in SMRTportal 2.3 (Pacific Biosciences). An aliquot of gDNA was also used to generate a library with Nextera XT kit and sequenced by Illumina MiSeq using a 300-bp paired-end strategy with MiSeq Reagent Kit v3 (Illumina, Inc. San Diego, CA, USA), according to manufacturer's instruction. The Illumina reads were trimmed with cutadapt (v1.14, min length 50 bp, quality cut-off 25, Martin, 2011) before mapping with Burrows-Wheeler Aligner (Li and Durbin, 2009) and for polishing the draft assembly by Pilon (Walker et al., 2014). Complete genome sequences were circularized using a genome assembly program (Gap4, Staden package, Bonfield et al., 1995), and overlapping ends were removed manually. Default parameters were used for all software unless otherwise noted. The closed genome was rotated to start at the *dnaA* gene. The genomic sequences were uploaded to NCBI and automatically annotated with NCBI Prokaryotic Genome Annotation Pipeline (PGAP) version 4.12. Bacteriocin genes from the genome were searched with the online tool BAGEL4 (http://bagel4.molgenrug.nl/), and prophages with PHAST and PHASTER (http://phast.wishartlab.com/, https://phaster.ca/). Genes encoding lytic enzymes were searched by translating the genome with ORF Finder (https://www.bioinformatics.org/sms2/orf_find.html) and comparing the protein sequences with NCBI Protein BLAST (https://blast.ncbi.nlm.nih.gov/Blast.cgi). Genes *lysL, lysP*, and *lysT* were manually curated for open reading frame regions. Conserved domains in proteins were analyzed with NCBI Conserved Domain Database (https://www.ncbi.nlm.nih.gov/Structure/cdd/wrpsb.cgi). For the prediction of signal peptides for protein secretion, Phobius, PrediSi, and SignalP-6.0 software were used (https://phobius.sbc.su.se/, http://www.predisi.de/, https://services.healthtech.dtu.dk/services/SignalP-6.0/).

### 2.4. LC-MS analysis of supernatant proteins

#### 2.4.1. Sample preparations

Total proteins of LAC460 culture supernatant were identified with liquid chromatography/mass spectrometry (LC-MS) at Meilahti Clinical Proteomics Core Facility, University of Helsinki, Finland. For the identification, supernatant proteins were precipitated with trichloroacetic acid (TCA) essentially as described by Sánchez et al. (2009). Briefly, 20 mg of sodium deoxycholate was added into 10 ml of o/n filtered LAC460 CFS and incubated for 30 min at 4°C, after which 600 μl of TCA was added and allowed to precipitate o/n at 4°C. Precipitated proteins were collected by centrifuging (9,300 *g*, 10 min, 4°C), the pellet was washed twice with 2 ml of chilled acetone, air-dried at RT, and dissolved in 200 μl of PBS (pH 7.4).

#### 2.4.2. LC-MS analysis

Further sample preparation, i.e., protein reductions and alkylations, proteolytic digestion, cleaning, drying, resuspension in 1% trifluoroacetic acid, sonication, and LC-MS analysis were performed according to the protocols previously described by Mäkelä et al. (2016). Three hundred nanograms of digested proteins were injected for LC-MS analysis. The peptides were separated by nanoAcquity UPLC system (Waters UK Ltd., Wilmslow, UK) equipped with a Symmetry C18 reverse phase trapping column (180 μm × 20 mm, 5 μm particles; Waters), followed by an analytical BEH-130 C18 reversed-phase column (75 μm × 250 mm, 1.7 μm particles; Waters) in a single pump trapping mode. The samples were then run in ion mobility-assisted data-independent analysis (HDMSE), in a Synapt G2-S mass spectrometer (Waters). Proteins were identified essentially according to the protocol described earlier (Mäkelä et al., 2016), except the phage lysin search in the present study was carried out against a self-made “database” of the six *L. lactis* LAC460 phage lysin sequences.

### 2.5. DNA techniques

DNA fragments for cloning, screening, and sequencing were amplified by PCR with Phusion High-Fidelity DNA polymerase (Thermo Scientific, Waltham, MA, USA) in Eppendorf Mastercycler (Eppendorf, Hamburg, Germany). PCR primers used are shown in Table 2. PCR products were purified with SanPrep Column PCR Product Purification kit (BBI Life Sciences, Shanghai, China), or extracted from agarose gel with SanPrep Column Gel Extraction kit (BBI Life Sciences). Plasmids were isolated with the SanPrep Column Plasmid Miniprep Kit (BBI Life Sciences). Plasmids and PCR products were sequenced by DNA Sequencing Service at the Institute of Biotechnology, University of Helsinki, Finland.

**Table 2:** PCR primers.

**Primer name**	**Use/target**	**Sequence^a^**
Erm F OE-LysL	*ermC*, overlap extension to H2 of *lysL* gene	ctaatcggtgtaATAATCGCATCCGATTGCAG
Erm F OE-LysP	*ermC*, overlap extension to H2 of *lysP* gene	tgatcgaacgacATAATCGCATCCGATTGCAG
Erm F OE-LysT	*ermC*, overlap extension to H2 of *lysT* gene	tgagtttagcgAATAATCGCATCCGATTGCAG
Erm R	*ermC, ermC*-*repAC* from pG^+^Host4	TCACAAAAAATAGGCACACG
Erm R OE-LysP	*ermC*, overlap extension to 347-bp fragment of *lysP* gene	ataggttgtctaTCACAAAAAATAGGCACACG
Rep F	*repAC* from pG^+^Host4	CGTTTCTGAGACGTTTTAGCG
Rep R	*repAC* and *ermC*-*repAC* from pG^+^Host4	GCTATTAATCGCAACATCAAACC
LysL H1 F	Upstream homologous fragment for deleting *lysL* gene	TCAACAACCGAGTAGCCGAG
LysL H1 R OE-H2	Upstream homologous fragment for deleting *lysL* gene	aactactaccgtCTAGGAATGACTCAAAGCCG
LysL H2 F OE-H1	Downstream homologous fragment for deleting *lysL* gene	agtcattcctagACGGTAGTAGTTCAGGGTTC
LysL H2 R OE-ermC	Downstream homologous fragment for deleting *lysL* gene	ggatgcgattatTACACCGATTAGTCCAGTAG
LysP H1 F	Upstream homologous fragment for deleting *lysP* gene	TTGAAATCCCAAGACCAATC
LysP H1 R OE-H2	Upstream homologous fragment for deleting *lysP* gene	aactgaataagcCAAGCATGGTTTCTCTCAGG
LysP H2 F OE-H1	Downstream homologous fragment for deleting *lysP* gene	aaaccatgcttgGCTTATTCAGTTCAATTAAACTCG
LysP H2 R OE-ermC	Downstream homologous fragment for deleting *lysP* gene	ggatgcgattatGTCGTTCGATCAAGCGTCTC
LysT H1 F	Upstream homologous fragment for deleting *lysT* gene	TCAAATCGCTGTGGTTCTCC
LysT H1 R OE-H2	Upstream homologous fragment for deleting *lysT* gene	gagtcattcctaACTCCCTGACTTGCTATTCG
LysT H2 F OE-H1	Downstream homologous fragment for deleting *lysT* gene	aagtcagggagtTAGGAATGACTCAAACTCGGA
LysT H2 R OE-ermC	Downstream homologous fragment for deleting *lysT* gene	ggatgcgattatTCGCTAAACTCAACTGGAGG
LysP-ins F OE-ermC	347-bp fragment for inactivating *lysP* gene	tattttttgtgaTAGACAACCTATGATCGTACAG
LysP-ins R	347-bp fragment for inactivating *lysP* gene	CTTTGAATGCCGATGATTGC

^a^Lowercase indicates overlap extension for OE-PCR.

T4 DNA ligase and T4 polynucleotide kinase were used as recommended by the manufacturer (Thermo Scientific). Ligation mixtures and plasmids were transferred into *E. coli* and *L. lactis* by electroporation with a Bio-Rad Gene Pulser device (Bio-Rad Laboratories, Hercules, CA, USA), essentially as described previously (Holo and Nes, 1989; Zabarovsky and Winberg, 1990).

### 2.6. Constructing the deletion and inactivation plasmids

Genes from the LAC460 chromosome were deleted by homologous recombination using thermosensitive plasmids. These integration plasmids harbored thermosensitive replication gene *repA* of pG^+^Host4, the erythromycin resistance gene *ermC*, and two 1-kb homologous fragments (H1, H2) upstream and downstream of the target gene. The H1 and H2 fragments from the LAC460 chromosome and the *ermC* gene from pG^+^Host4 were joined together by OE-PCR. The fragment fusions were phosphorylated and ligated with *repAC* amplicon from pG^+^Host4, and the ligation mixtures were electroporated into *E. coli* TG1. Clones were screened using PCR; the correct plasmids were isolated and electroporated into LAC460. Gene deletions were achieved by following the thermosensitive single- and double cross-over procedures described by Biswas et al. (1993). Correct knock-outs were distinguished from wild types by PCR confirmations with H1F-H2R primers.

For the inactivation of the 732-bp peptidase M23 gene *lysP*, a 347-bp fragment in the middle of the gene (from 72 to 418 bp) was used for the integration of the plasmid into the chromosome. The fragment was amplified by PCR, phosphorylated, and ligated with *ermC*-*repAC* from pG^+^Host4, after which the same abovementioned procedure for transformations and thermosensitive integration was followed until a single cross-over integrant strain was obtained. The correct insertion of the plasmid into the *lysP* gene was confirmed by PCR. The insertion splits the gene into two parts of 418 and 661 bp, the latter lacking a start codon.

### 2.7. Turbidity reduction assay

The capacity of the LAC460 CFS to lyse the host cells was tested with cell resuspensions in PBS. *Lactococcus* test strains LAC460, LM0230, MG1614, and N8 were first cultured overnight in 4 ml M17G. Then, the cultures were centrifuged (5,000 *g*, 10 min), and the cell pellets were washed with PBS and resuspended in 4 ml of PBS. Honeycomb microtiter plate wells were filled with 270 μl of the cell suspensions and 30 μl of either filtered or pasteurized (75°C, 10 min) LAC460 CFS. Cell disruption was then determined by measuring every hour the optical density at 600 nm wavelength at 30°C in Bioscreen C (Growth Curves Ltd., Helsinki, Finland). Five parallel measurements with each strain were done.

### 2.8. Cell leakage test

The ability of the LAC460 lysins to make target cells leaky without actual cell lysis was determined by measuring the release of cytosolic enzyme lactate dehydrogenase (LDH) from cells treated with different lysins. To speed up the lytic action, concentrated CFSs were used. *Lactococcus* test strains LAC460, LM0230, and MG1614 were cultured overnight in 10 ml M17G broth, cells were pelleted, washed with PBS, and resuspended in 7.5 ml PBS. Then, 135 μl cell suspensions were mixed with 15 μl of the concentrated CFSs or commercial lysozyme (100 mg/ml; Merck) as a positive control in Eppendorf tubes, and incubated at 30°C for 1 h. The cells were removed by centrifuging, and 100 μl of clear liquid was transferred onto a 96-well flat-bottom transparent microplate (Sarstedt, Nümbrecht, Germany). The presence of LDH in the samples was determined with a CyQUANT LDH cytotoxicity assay kit according to the manufacturer's instructions (Invitrogen, Waltham, MA, USA). Briefly, 50 μl of the kit reaction mixture was added to 100 μl samples in microplate wells, incubated for 1 h at RT, and the color reaction was stopped with 50 μl of the kit stop-solution. The absorbance at 490 and 680 nm was measured with a Tecan Spark plate reader (Tecan Group Ltd., Männedorf, Switzerland). All measurements were done in triplicate.

### 2.9. Zymographic analysis

The cell lysis by LAC460 prophage lysins was investigated in zymogram gel, essentially according to the protocol by Buist et al. (1995). Concentrated CFSs (5–7 μl) were mixed with one volume of 2 × Laemmli sample buffer (Bio-Rad Laboratories) supplemented with 5% of β-mercaptoethanol, and run in regular 10% SDS-PAGE mini-gels containing 0.3% of *Lactococcus* indicator cells of the strains LAC460, LM0230, or MG1614. The gels were run in conventional Tris-glycine-SDS buffer in Bio-Rad Mini-Protean 3 electrophoresis system at 4°C with 100 V (approximately 40 mA) for 30 min, after which the volts were raised up to 150 V (approximately 40 mA) and the run continued for about 2 h. After electrophoresis, gels were washed three times for 10 min with distilled water. Gels were incubated with gentle shaking in 100 ml of refolding buffer (25 mM Tris, 1% Triton X-100, pH 7) for approximately 40 h including three buffer changes. Gels were stained with 0.1% methylene blue in 0.01% KOH for 3 h and finally destained with distilled water.

### 2.10. Bacteriocin production at different growth stages

Bacteriocin quantification at different growth stages was performed according to Nilsen et al. (2003) with some modifications. Cells from 50 ml overnight cultures of *Lactococcus* indicator strains LM0230 and MG1614 were harvested (5,000 g, 10 min), washed with PBS, resuspended in 50 ml PBS, and kept at 4°C until the end of the experiment. Two hundred microliters of *L. lactis* LAC460 overnight culture was inoculated into 200 ml M17G and the culture was incubated at 30°C. Two milliliters of samples were taken every hour for 16 h. Of the 2-ml sample, 1 ml was used for optical density measurement (OD_600_) for growth curve, and 1 ml for bacteriocin quantification. For the latter, the sample was first centrifuged (5,000 *g*, 10 min) and filtered through a sterile 0.22 μm syringe filter. This filtered LAC460 CFS was diluted in PBS to 1/2, 1/4, 1/8, 1/16, and 1/32. A total of 150 μl of the CFS dilutions was added into 150 μl indicator cell suspension in a honeycomb microtiter plate. Indicator cell lysis was monitored for 9 h by measuring OD_600_ every hour using Bioscreen C (Growth Curves Ltd.). All measurements were carried out in triplicate. The bacteriocin unit was defined as the reciprocal of the supernatant dilution decreasing OD_600_ of the indicator suspension by 50% in 4 h at 30°C. The limit of 4 h was chosen as a reasonable incubation time to see the differences in bacteriocin concentrations.

## 3. Results

### 3.1. Identification of an antimicrobial strain of *Lactococcus lactis*

**Figure 1:**
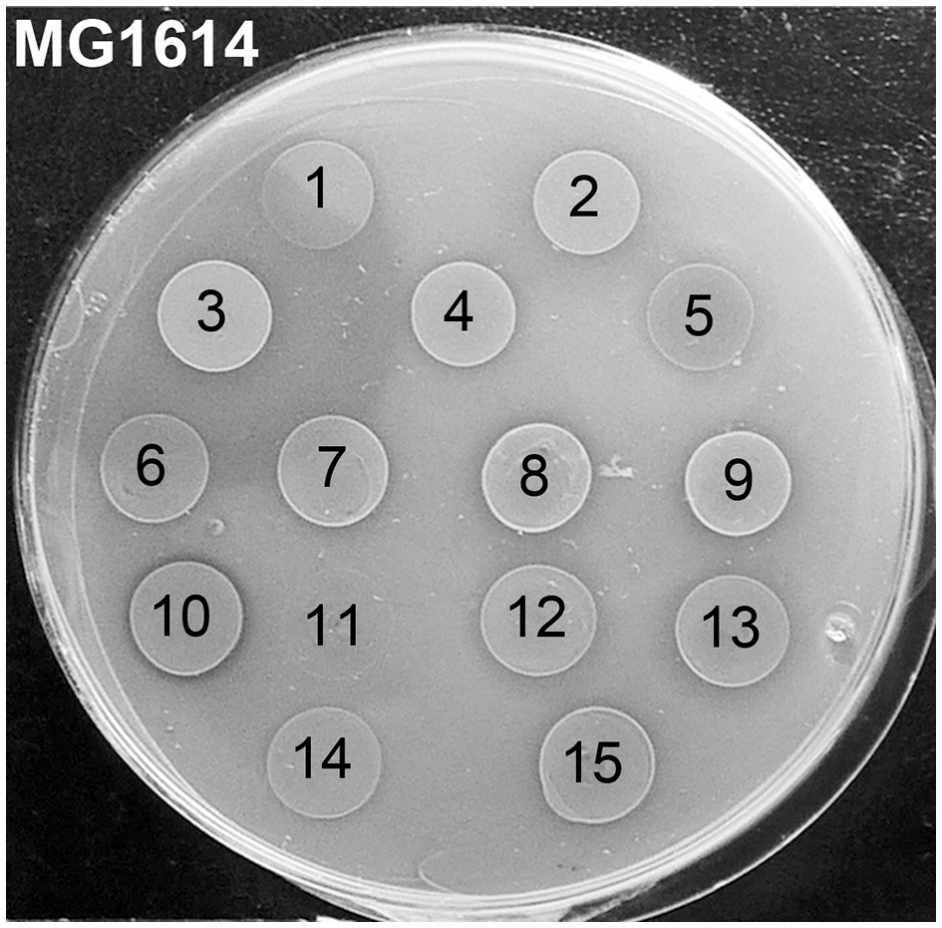
Antimicrobial activity of 15 *Lactococcus* isolates from idli batter against *Lactococcus cremoris* MG1614 indicator strain. Ten microliters of o/n cultures of the isolates were spotted on the indicator lawn. The isolate number 3 with an inhibition zone was chosen for further studies and given the name LAC460. The isolate number 10 with a narrow clear inhibition zone was found to be a nisin Z producer. Isolates: 1, *L. cremoris* FA72; 2, *L. lactis* FA73; 3, *L. lactis* FA74 = LAC460; 4, *L. lactis* IA71; 5, *L. taiwanensis* IA72; 6, *L. lactis* LA71; 7, *L. lactis* LA72; 8, *L. lactis* L71; 9, *L. lactis* L4Y2; 10, *L. lactis* L471; 11, *L. lactis* L4M2; 12, *L. garvieae* FY2; 13, *L. garvieae* F71; 14, *L. garvieae* F4Y2; 15, *L. lactis* F471.

In our previous study (Colombo, 2016), we isolated and identified 76 strains of lactic acid bacteria (15 *Lactococcus* spp., 11 *Leuconostoc* spp., and 50 *Weissella* spp. isolates) from spontaneously fermented idli batter made of ground rice and different beans. The first aim of the current study was to analyze whether these idli isolates possess antimicrobial activities. The strains and their CFSs were first tested on agar plates with three indicator strains. From idli batter made of rice and faba beans, we found an *L. lactis* strain showing strong activity against *L. cremoris* MG1614 (Figure 1), but being inactive against *L. monocytogenes* V 2872 and *Micrococcus luteus* NCIMB 8166. The strain was chosen for further analyses and given the name LAC460.

Then, the antimicrobial spectrum of LAC460 was determined through spot-on-lawn assay using both cells and filtered CFS of LAC460 o/n culture. We tested Gram-positive indicator bacteria from the genera *Bacillus, Enterococcus*, lactobacilli, *Lactococcus, Leuconostoc, Listeria, Micrococcus, Staphylococcus, Streptococcus*, and *Weissella*. Tested indicator strains and their susceptibilities are listed in Supplementary Table 1. *Lactococcus* was the only genus sensitive to the antimicrobial substance both from the cells and CFS (17 sensitive out of 34 strains tested). It could be concluded that this substance is specific with a very narrow target range, as only the closest relatives were killed.

### 3.2. Characterization of the antimicrobial substance of LAC460

Temperature, pH, and proteolytic enzymes are known to affect proteinaceous compounds, and thus they also affect bacteriocins. To assess, whether the antimicrobial substance from the strain LAC460 was a bacteriocin, CFS was treated with proteases, kept at different temperatures, or adjusted to different pH, after which the antimicrobial activity was tested on *L. cremoris* MG1614 indicator plate. The antimicrobial activity was lost after incubation with proteases, such as proteinase K, demonstrating that the antimicrobial substance was a protein. Therefore, according to the definition of bacteriocin, the substance could be called bacteriocin. The bacteriocin did not tolerate low or high pH, as the inhibition zone on the indicator plate weakened when the supernatant was kept for 2 h at pH 4 or pH 10, and the activity was permanently lost at pH 3 and pH 11. Overnight storage at 4°C or −20°C did not seemingly affect the activity, but the bacteriocin was found to be sensitive to heat. It tolerated 60 min at 45°C with no apparent reduction in activity, but when the CFS was kept at 53°C for 15 min, the activity vanished completely. The sensitivity to heat suggested that the bacteriocin could belong to the class III bacteriocins, i.e., heat-labile bigger proteins.

### 3.3. Genome sequencing: searching for the genes encoding lytic enzymes

As seen in Figure 1, the inhibition zone caused by LAC460 is formed of a narrow clear halo and a wider hazier zone. According to phase-contrast microscopy analysis, the hazy halo mainly contained cell debris, i.e., lysed cells. Hence, as the bacteriocin seemed to be a lytic enzyme, either autolysin, phage lysin, or bacteriolysin, our aim was to look for the genes encoding lytic enzymes from the genome sequence. The whole genome of LAC460 was sequenced, revealing a 2 426 597 bp chromosome without plasmids. The complete genomic sequence has been deposited in GenBank and is available under the accession number CP059048. The genome was analyzed with the BAGEL4 online tool for the presence of bacteriocin genes. No obvious bacteriocin or bacteriolysin genes were found. Then, the genome was translated with ORF Finder, and the resulting protein sequences were analyzed using NCBI Protein BLAST to identify lytic enzymes. Altogether 20 lysins, probable autolysins and phage lysins, were identified from the LAC460 proteome (Supplementary Table 2). According to the online prophage finder tools PHAST and PHASTER, LAC460 carries 4 prophage regions, namely PLl460-1 (10.8 kb, position 531,499–542,307 in genome sequence), PLl460-2 (18.7 kb, position 1,656,522–1,675,285), PLl460-3 (62.6 kb, position 1,695,599–1,758,259), and PLl460-4 (18.1 kb, position 2,132,191–2,150,384), of which PLl460-3 is possibly intact, and the other three are incomplete defect prophages. Based on comparisons to NCBI GenBank, there are no lysin genes in PLl460-4, whereas the other three carry two lysin genes each, apparently one endolysin and one VAL. As prophage lysins are known to be expressed and secreted in lactococci, our next aim was to determine if they are also found in the LAC460 culture supernatant.

### 3.4. LC-MS analysis of supernatant proteins for prophage lysins

**Figure 2:**
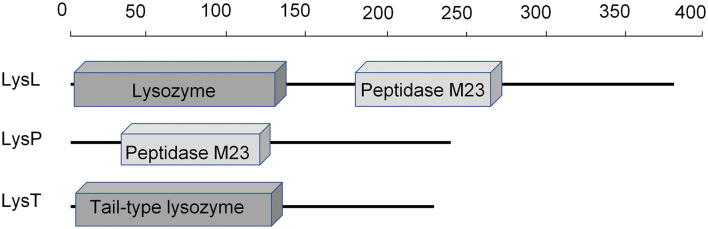
Locations of recognized enzymatically active domains in the three phage lysins found in the LAC460 culture supernatant. The C-termini do not show similarity to known conserved domains. Signal peptides for secretion were not predicted from any of the three proteins. The scale indicates the number of amino acid residues.

As genome sequencing did not reveal any obvious bacteriocin gene, we aimed to search for phage lysins in the culture supernatant. In order to find out whether the prophage lysins were expressed and secreted, total supernatant proteins from o/n culture were identified using LC-MS and compared with the sequences of the six LAC460 phage lysins. The analysis revealed that three phage lysins were present in the supernatant. According to NCBI sequence comparison BLAST and Conserved Domain search, the three phage lysins in the supernatant were a 385-aa lysozyme+peptidase M23 (LysL, in defect prophage PLl460-1, locus tag H0A38_RS02765 in genome sequence), a 243-aa peptidase M23 (LysP, in defect prophage PLl460-2, locus tag H0A38_RS07975 in genome sequence), and a 233-aa phage tail-type lysozyme (LysT, in possibly intact prophage PLl460-3, locus tag H0A38_RS08355 in genome sequence). Amino acid sequences of the three lysins are presented in Supplementary material 1. According to online signal peptide prediction tools, none of the three phage lysins carry signal peptides for secretion. Based on the NCBI Conserved Domain Database, the C-termini of about 87–115 aa in all three enzymes are of unknown function without homology to conserved domains (Figure 2), suggesting that the enzymes may contain C-terminal cell wall binding domains, presumably explaining the high specificity of the bacteriocin. To sum up, the LAC460 culture supernatant contains three prophage lysins, of which one or more could be responsible for the observed lytic bacteriocin activity.

### 3.5. Deletion and inactivation of the three phage lysin genes

To find out which of the three lysins, if any, was responsible for the bacteriocin action, we aimed to delete the lysin genes from the chromosome and make single, double, and triple knock-out strains. A conventional double cross-over method with a temperature-sensitive plasmid vector was chosen for the deletion strategy. Two fragments flanking the lysin genes were cloned with the *ermC* and the thermosensitive *repAC* (TS*repAC*) genes from the vector pG^+^Host4. The constructs were made in *E. coli*, after which the plasmids were introduced into LAC460 and the gene deletions were obtained after the two cross-overs and vector elimination. Unfortunately, despite many different attempts and approaches, including commercial DNA construction service (Genscript, Piscataway, NJ, USA), the clonings for the plasmid aiming to delete the peptidase M23 (LysP) gene *lysP* were unsuccessful. Possibly the regions flanking the lysin gene were lethal in *E. coli* and *Lactococcus* when cloned in a plasmid, and thus that construct could not be obtained. Instead, we then aimed to inactivate the gene encoding LysP by plasmid insertion. For this, a 347-bp fragment from the middle of the *lysP* gene was cloned with the *ermC*-TS*repAC* fragment, electroporated into LAC460, and integrated into the *lysP* gene.

**Figure 3:**
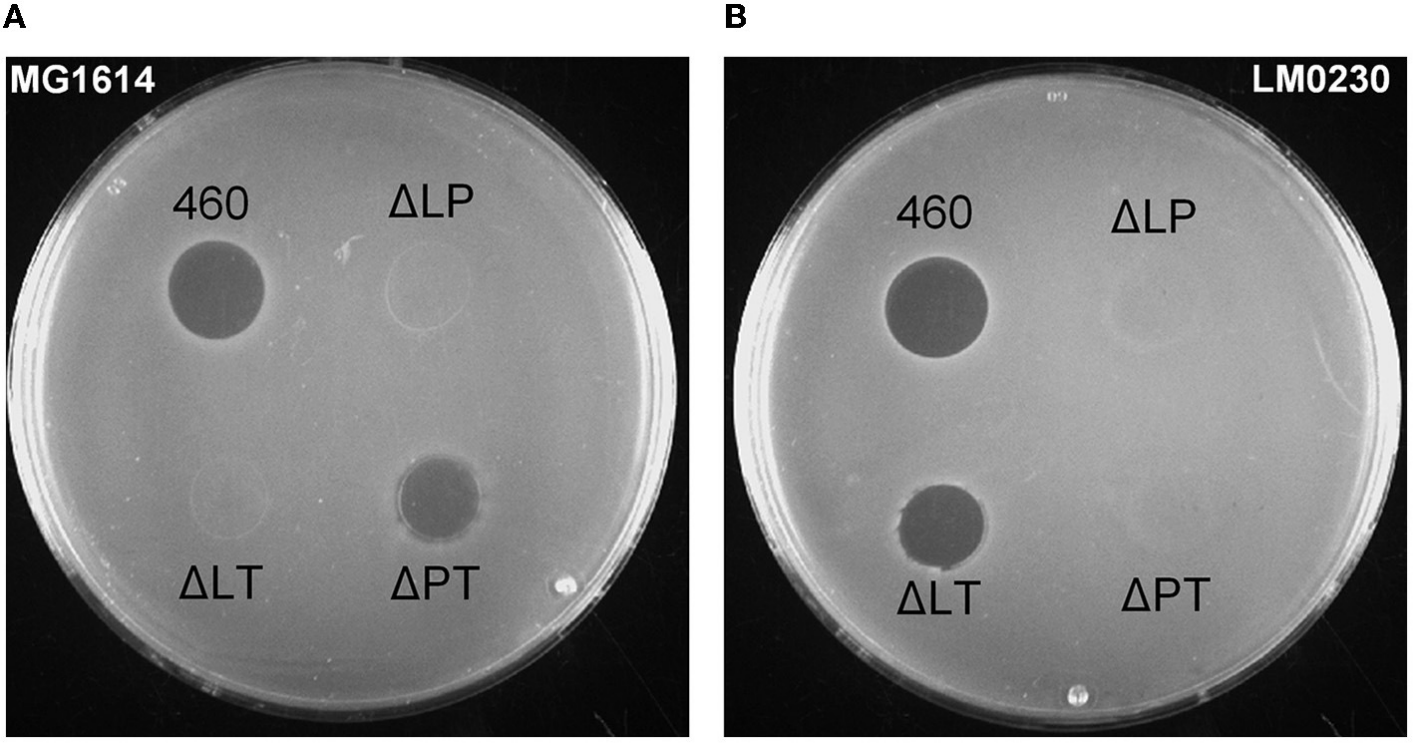
Inhibition zones of double lysin knock-out strains of LAC460 on *L. cremoris* MG1614 **(A)** and *L. lactis* LM0230 **(B)** indicator lawns. Concentrated CFS from o/n cultures of the wild-type LAC460 and the knock-out strains were spotted on the indicator lawns. 460, *L. lactis* LAC460; L, LysL lysozyme+peptidase M23; P, LysP peptidase M23; T, LysT tail-type lysozyme. LAC460 kills both indicators. Based on the knock-out strain halos, LysL only kills MG1614, LysP only kills LM0230, and LysT does not kill either of the indicators.

The antimicrobial activity of the deletion and inactivation strains was tested with concentrated CFSs on indicator plates with 34 *Lactococcus* strains. As can be deduced from the inhibitions on *L. cremoris* MG1614 and *L. lactis* LM0230 plates by the double knock-out strains shown in Figure 3, the LysL lysozyme+peptidase M23 was active against MG1614, but not against LM0230. On the contrary, the LysP peptidase M23 killed LM0230, but not MG1614. LysT tail-type lysozyme did not kill any of the indicators, concluding that LysL and LysP were the ones responsible for the bacteriocin action. All 17 LAC460-sensitive *Lactococcus* strains were killed either by LysL or LysP, but never by both (Supplementary Table 1).

### 3.6. Turbidity reduction assay

**Figure 4:**
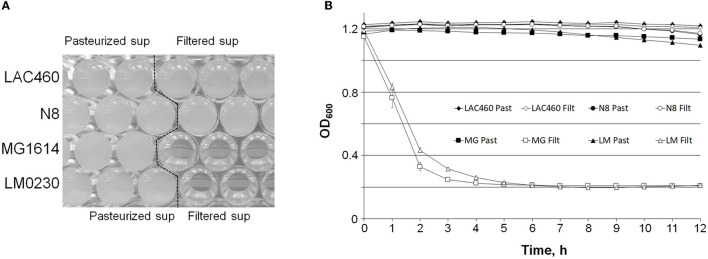
Turbidity reduction assay of *Lactococcus* indicator cells by LAC460 culture supernatant. Filtered or pasteurized CFS of o/n LAC460 culture was added into suspensions of four *Lactococcus* strains, and the cell lysis was determined as the decrease of OD_600_. **(A)** Honeycomb microtiter plate with *Lactococcus* cell suspensions after 24 h with pasteurized or filtered LAC460 supernatant, demonstrating complete clearance of LM0230 and MG1614 suspensions by filtered LAC460 CFS. **(B)** OD_600_ measurements of the suspensions, showing the lysis of LM0230 and MG1614 by filtered LAC460 CFS, as well as the resistance of LAC460 to its own prophage lysins. Error bars represent the standard deviation of five parallels.

The natural function of prophage lysins is to degrade the host cell wall. Because the LAC460 CFS did not make a halo on the LAC460 indicator plate (result not shown), we tested whether the LAC460 prophage lysins lyse the host in the cell suspension. We used four *Lactococcus* indicator strains in the autolysis test: the host LAC460, the sensitive strains LM0230 and MG1614, and the resistant strain N8. Filtered or pasteurized LAC460 CFS was added into indicator cell suspension in PBS, and the cell lysis was monitored as a decrease of OD_600_. Since the lysins are heat sensitive, they are only active in filtered CFS, but inactive when pasteurized. The suspensions of the sensitive strains LM0230 and MG1614 became totally clear in approximately 3–4 h, as also seen as a strong drop of OD_600_ (Figure 4). Quite the opposite, the cell suspensions of the resistant strain N8 and the host LAC460 remained turbid, and their OD_600_ did not significantly decrease in 24 h. Therefore, it can be concluded that LAC460 was not lysed and that the strain is resistant to autolysis by its own prophage lysins.

### 3.7. Release of cytosolic enzymes

**Figure 5:**
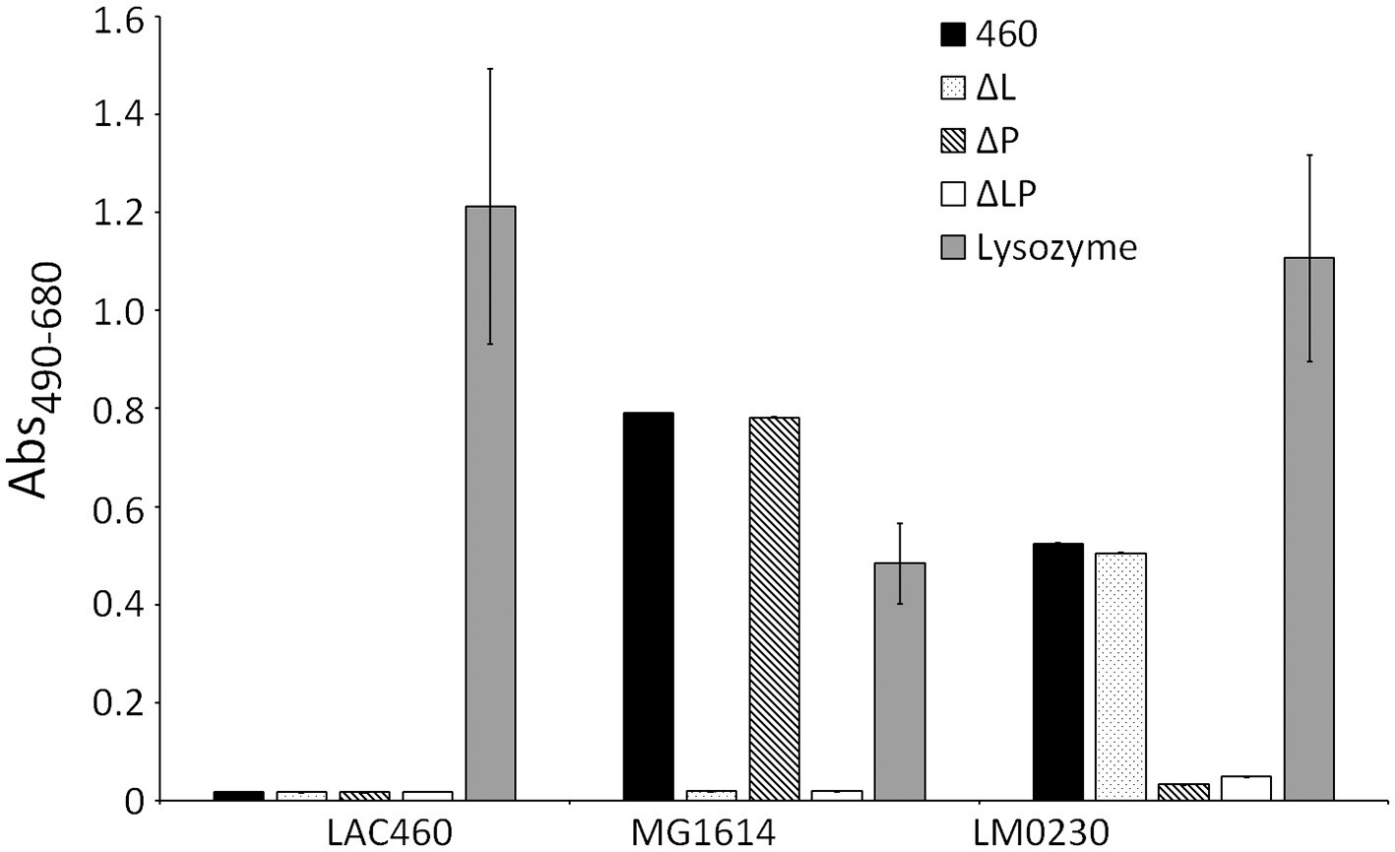
Release of LDH from CFS-treated *Lactococcus* strains LAC460, MG1614, and LM0230. *Lactococcus* strains were incubated with concentrated CFS from wild-type LAC460, its LysL and LysP knock-out strains, or with commercial lysozyme. Leakage of cytosolic LDH enzyme demonstrates breaking of the cells caused by LAC460 phage lysins. LDH was released from the strain MG1614 with lysozyme and with LysL containing CFSs. From the strain LM0230, LDH was released with lysozyme and with LysP containing CFSs. From LAC460, LDH was only released with lysozyme, confirming the strain's resistance to LysL and LysP. Error bars represent the standard deviation of three parallels.

Even though LAC460 was not lysed by its own phage lysins, it is possible that the lysins degrade the cell walls only locally, without a significant decrease in optical density, but damaging the cells and leading to cell leakage. To find out that, we tested whether the lysins cause the release of the cytosolic enzyme lactate dehydrogenase (LDH) from cells to the environment by using an LDH cytotoxicity assay. The release of LDH from the *Lactococcus* strains LAC460, LM0230, and MG1614 was determined with concentrated CFSs of the wild-type LAC460 and the LysL/LysP knock-out strains. Commercial lysozyme was used as a control. Figure 5 shows that CFS of Δ*lysLP* did not cause significant cell leakage in any strains, whereas all test strains released LDH when treated with commercial lysozyme. As expected, LDH was also released from the strain LM0230 with LysP containing CFSs, and from MG1614 with LysL containing CFSs. None of the CFSs caused LDH release in LAC460, again signifying that LAC460 phage lysins do not degrade the host cell wall.

### 3.8. Zymographic analysis

**Figure 6:**
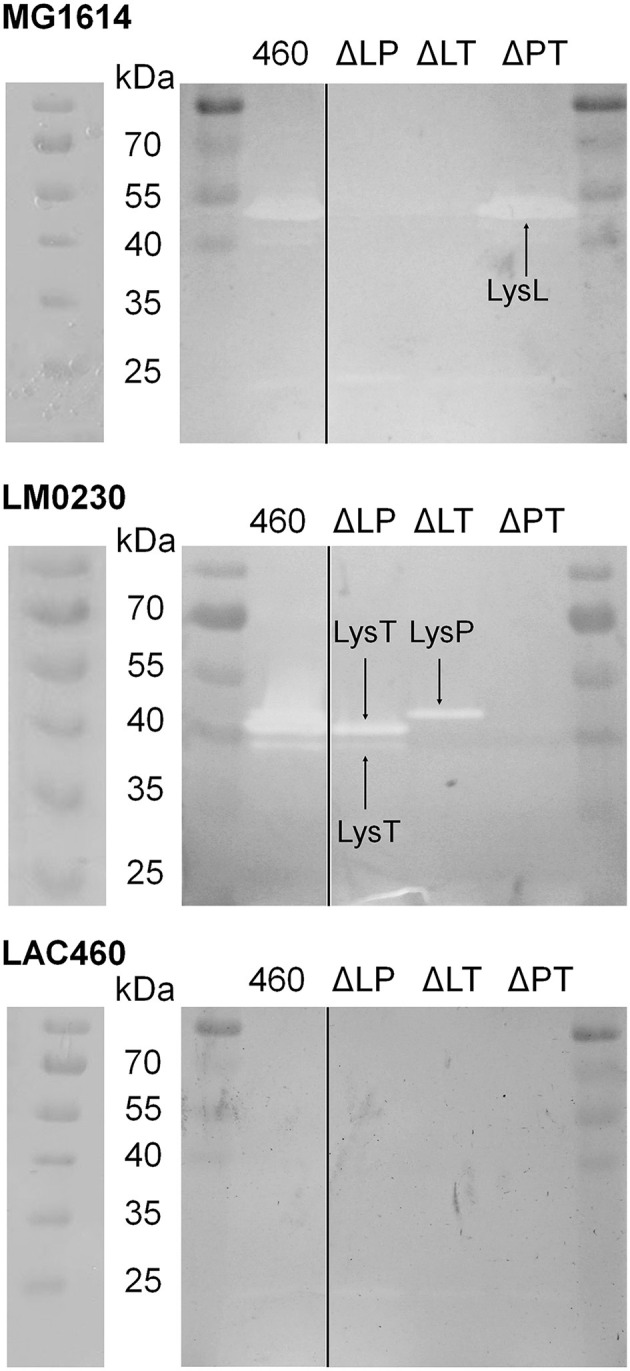
Zymographic analysis of LAC460 culture supernatant proteins against *Lactococcus* substrate cells of the strains LM0230, MG1614, and LAC460. Concentrated supernatants of the wild-type LAC460 and the double lysin knock-out strains (ΔLP, ΔLT, and ΔPT) were run in SDS-PAGE, allowing the enzymes to renaturate and lyse the substrate cells, followed by gel staining. Based on the obtained and missing bands from the knock-out strain CFSs, LysL only lyses MG1614 cells, whereas LysP and LysT only lyse LM0230 cells. The two LysT bands indicate possible two forms of LysT. None of the three phage lysins lyse LAC460 cells. M, protein size marker PageRuler. Left gels, the same gels photographed immediately after electrophoresis, showing the PageRuler marker bands clearer.

As all the three studied lysins, LysL, LysP, and LysT, may be virion-associated lysins (VALs), being part of the phage structure, it is possible that in the LAC460 culture supernatant, they do not exist as single proteins, but as complexes with other phage proteins, or even as PTLBs. In order to find out whether the three lysins are able to lyse cells individually as single proteins, and to observe the actual function and lysis of each LAC460 phage lysin, a zymographic analysis was conducted after protein separation in denaturing SDS gel, in which protein complexes dissociate into monomers retaining only their primary structure. Concentrated CFSs from LAC460 and the double knock-out strains of the three phage lysins (LysL, LysP, and LysT) were run in SDS-PAGE containing *Lactococcus* cells of the indicator strains LAC460, LM0230, or MG1614 as substrate, followed by renaturation of the proteins and staining the gels. The zymogram gels are shown in Figure 6. No phage lysin bands could be seen in the gel with LAC460 cells, ultimately concluding that the three phage lysins do not lyse their host. In the gel with MG1614 cells, a strong band at between 40 and 50 kDa is seen with LysL containing CFSs, i.e., samples from wild-type LAC460 and Δ*lysPT*. The band is missing in samples from strains with *lysL* deletion, confirming the lytic action of LysL (43 kDa) against MG1614. LysP or LysT did not give bands in MG1614 gel, obviously because they do not break its cell wall. In the gel with LM0230 cells, the double knock-out strains Δ*lysLT* and Δ*lysLP* gave altogether three bands of around 40 kDa, representing the activity of LysP and LysT; the biggest band is LysP, and two smaller ones are LysT. Hence, even though LysT had neither killed nor made indicator cells leaky, it was active lysin in the zymogram, showing its function as a lytic enzyme and its ability to degrade cell walls. The double knock-out strain Δ*lysPT* did not give bands, showing that LysL does not degrade the cell wall of LM0230.

A few faint smaller bands can also be seen in the zymogram gels. We do not know, which enzymes are responsible for these other bands, but it would not be surprising if some autolysins are present in the CFSs. However, as shown in other experiments, those enzymes are not the cause of the antimicrobial activity of LAC460. In conclusion, the zymographic analysis showed the individual lytic action of the two phage lysin-like bacteriocins LysL and LysP, as well as the third phage lysin present in LAC460 supernatant, the putative tail lysozyme LysT.

### 3.9. Bacteriocin production at different growth stages

**Figure 7:**
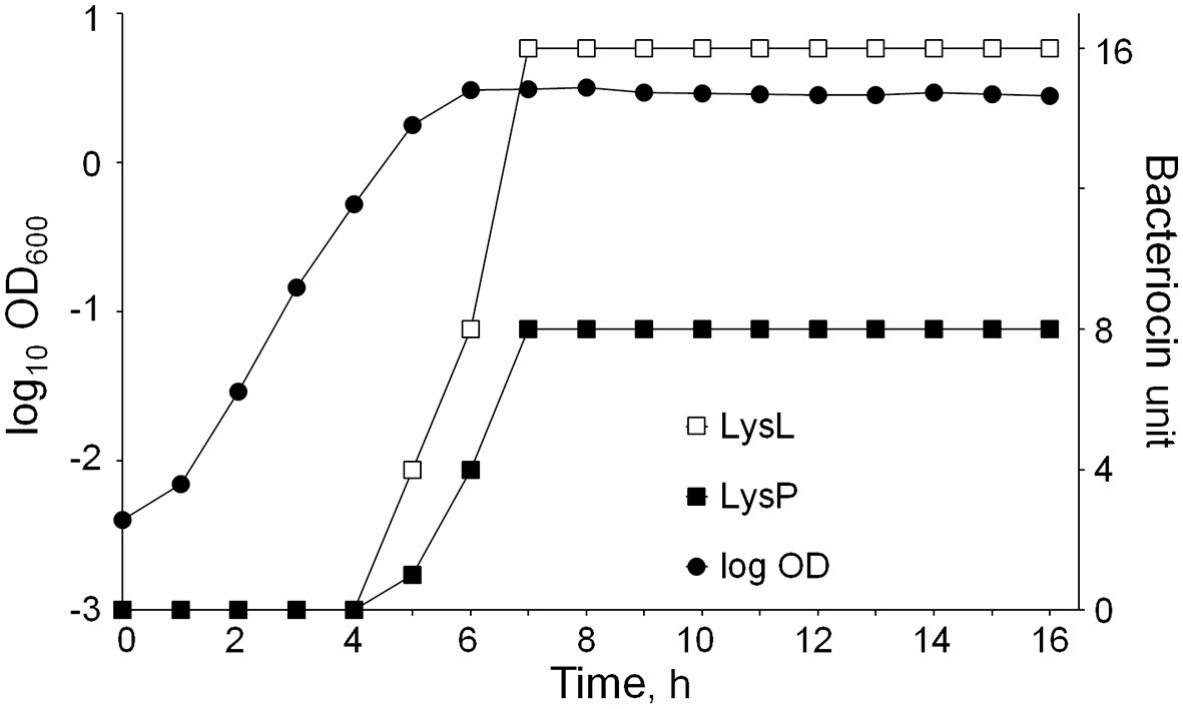
*L. lactis* LAC460 growth curve and lysin production at different growth stages. Lytic activity increases after 4 h and reaches its maximum at 7 h. Hence, lysins are produced between 4 and 7 h after inoculation, representing the later logarithmic growth phase and early stationary phase. Lysin activity did not increase in the later stationary phase.

Common to phage lysins, the three LAC460 lysins seemed not to contain signal peptides for secretion. Their secretion may be based on holin action, or simply on cell autolysis in the stationary/death phase releasing intracellular proteins into supernatant. To find out if the latter way of “secretion” is the case here, we aimed to determine at which growth stage these lysins are secreted. LAC460 was cultured for 16 h, and every hour the OD_600_ was measured, and bacteriocin activity was quantified by using suspensions of the *Lactococcus* indicator strains LM0230 and MG1614. With MG1614, the LysL activity, and with LM0230, LysP activity could be determined. The lysins were found to be produced in the late log phase and early stationary phase, between 4 and 7 h after inoculation (Figure 7). Bacteriolytic activity did not increase after 7 h, which excludes the possibility of protein release through natural cell autolysis in the death phase. The experiment was continued overnight, and the last measurements were done 31 h after inoculation. The bacteriocin activities did not significantly increase during the extra 15-h incubation (results not shown).

## 4. Discussion

In this study, we present a wild-type *L. lactis* strain producing prophage lysins, which act like bacteriocins and kill other lactococci. Three prophage lysins, namely LysL, LysP, and LysT, were found from the LAC460 culture supernatant. By knock-out mutants, LysL and LysP from defective prophages were shown to be responsible for the antimicrobial activity. A few studies have previously shown the secretion of prophage lysins by wild-type lactococci (Pillidge et al., 2002; Visweswaran et al., 2017). However, in those studies, the lysins functioned as autolysins targeting the host cell wall and contributing to cell autolysis. Even though secreted prophage lysins in wild-type strains have not been reported to have antimicrobial bacteriocin-like activity, many phage lysins have been produced in heterologous bacteria as antimicrobials (Gaeng et al., 2000; Rodríguez-Rubio et al., 2012; Gervasi et al., 2014; Grabowski et al., 2021; Chandran et al., 2022). Due to their target specificity, foreign lysins do not affect the heterologous producer host but act as antimicrobials against other bacteria. Similar to the heterologous lysins, exogenously added LAC460 phage lysins in the present study did not lyse their host cells in suspension or in zymogram gel, and not even caused leakage of intracellular proteins from LAC460, and consequently, LysL and LysP could behave like antimicrobial proteins. The mechanism of the LAC460 resistance to its own phage lysins is not known. It may be due to the four prophage sequences on its chromosome, as prophages are known to provide protection against infection by similar phages (Jurado et al., 2022). More precisely, based on studies about the interactions of cells and phages or phage lysins, the resistance is likely to be related to cell surface structures and changes in them. For instance, increased cell wall cross-linking has been suggested to be the reason for increased resistance to streptococcal phage lysin LytA (DeBardeleben et al., 2014). Moreover, differences in cell wall polysaccharides have been shown to determine the phage sensitivity in *Lactococcus* spp. (Ainsworth et al., 2014). Strains of *Lactococcus* spp. can be classified into four major cell wall polysaccharide genotypes, determining the sensitivity to different phages (Lavelle et al., 2021). Yet, studies about binding targets for phage lysins are quite scarce. At least wall teichoic acids have been shown to be involved in the binding of phage endolysins to *L. monocytogenes* peptidoglycan (Eugster et al., 2011; Eugster and Loessner, 2012).

All the abovementioned cell surface polymers and their rearrangements result in high specificity of *Lactococcus* phages; one *Lactococcus* strain is susceptible only to some phages, and resistant to many others (Oliveira et al., 2018). This fits well with our results about the antimicrobial range of LysL and LysP. As shown in Supplementary Table 1, half (17/34) of the tested *Lactococcus* indicator strains were sensitive to LAC460 culture supernatant, and of those 17 susceptible strains, 11 were sensitive to LysL and 6 to LysP. Hence, similar to phages, also phage lysins are specific with a narrow target range, probably because they bind to specific receptors, which structures vary between strains. Only a little is known about cell surface structures or prophage sequences of the 34 *Lactococcus* spp. indicator strains tested in this study, and therefore further studies would be needed to find reasons for LysL and LysP sensitivity and the resistance mechanism.

It is not fully certain, whether LysL and LysP are endolysins or VALs. Genes *lysL* and *lysP* are not located next to holin genes, though not too far either, < 900 bp away. Yet, the other lysin genes in the same prophages (locus tag H0A38_RS02785 in PLl460-1 and H0A38_RS07955 in PLl460-2) are located adjacent to holin genes in the same direction, making them better candidates for endolysins. A clearer case is the third phage lysin present in the LAC460 culture supernatant, the phage tail-type lysozyme LysT, which is most likely a VAL, as in the prophage it is located about 20 kb away from a holin gene. The other lysin gene in the LysT carrying PLl460-3 prophage (locus tag H0A38_RS08230) is likely to encode an endolysin as the gene is located next to a holin gene, and the encoded protein is predicted to contain a *sec*-dependent signal peptide for secretion and a LysM motif for cell wall binding.

Thus far, all bacteriocins characterized from lactococci belong to either Class I, such as nisin (Gross and Morell, 1971), or Class II, such as lactococcin G (Nissen-Meyer et al., 1992). Lactococci have never been shown to produce Class III bacteriocins, heat-sensitive large antimicrobial lytic or non-lytic proteins. It has been proposed that larger antimicrobial proteins should not be called bacteriocins at all (Cotter et al., 2013; Soltani et al., 2021). However, according to the traditional definition of bacteriocins, i.e., ribosomally synthesized antimicrobial proteins produced by bacteria to inhibit the growth of similar or closely related bacterial strains, LysL and LysP from LAC460 could be regarded as bacteriocins. Another special type of class III bacteriocins is the PTLBs, large complexes consisting of phage structural proteins having antimicrobial activity (Scholl, 2017). As LysL and LysP were clearly of prophage origin, similar to PTLB, they could be regarded as a new type of antimicrobial proteins, namely phage lysin-like bacteriocins. In addition to PTLB and lysins, many other phage proteins are also toxic to bacteria. It has been suggested that the domestication of prophages or phage genes for the bacteria's own benefit is actually a common feature (Bobay et al., 2014). Not only lactococci secrete prophage lysins as autolysins (Pillidge et al., 2002; Visweswaran et al., 2017) but also the order Caulobacterales have domesticated a phage lysin by detoxification, resulting in attenuated lysin playing a role in cell morphology (Randich et al., 2019). Furthermore, some phage genes or gene clusters encode proteins, which are regarded as bacteriocins, for instance, sublancin from *B. subtilis* prophage (Oman et al., 2011). Yet, *L. lactis* LAC460 presented in this study is the first reported bacterial strain using prophage lysins as bacteriocins.

Endolysins of phages targeting Gram-positive bacteria are modular enzymes consisting of two main domains: usually N-terminal enzymatically active domain (EAD) that hydrolyzes peptidoglycan (PG), and usually C-terminal cell wall binding domain (CBD) (Abdelrahman et al., 2021). The effective hydrolytic activity of phage lysin requires the binding of the enzyme to the cell wall. While the EADs may be general cell wall hydrolyzing enzymes, such as muramidases able to hydrolyze the PG of different bacteria, the CBDs often show high specificity to the phage target strain and are thus responsible for the specificity of the lysin (Gómez-Torres et al., 2018; Abdelrahman et al., 2021). According to the conserved domain analyses, the N-termini of the lysins T, P, and L carried EADs lysozyme, peptidase, and lysozyme+peptidase, respectively. On the contrary, the C-termini (87 aa of lysin T, 115 aa of lysins P and L) did not show homology to conserved domains. Even though it was not experimentally evidenced in this study, the high specificity of the lysins suggests that they contain specific CBDs in the C-termini. The last 78 amino acids of LysP and LysT are identical (Supplementary material 1), suggesting that these two lysins would target the same receptor on the cell wall. Even though VALs are sometimes claimed not to contain CBDs (e.g., Chandran et al., 2022), there is also a report about the identification of a cell binding region in a VAL enzyme (Takác and Bläsi, 2005). Therefore, it cannot be excluded that the apparent VAL LysT would carry a CBD for specific cell wall binding.

As it is not fully clear whether the LAC460 phage lysins are endolysins or VALs, it is possible that they exist as complexes with other phage proteins. To find out, whether the lysins are able to lyse target cells as individual enzymes, a zymographic analysis was conducted after protein separation in denaturing gel. All three lysins, LysL, LysP, and LysT, could be identified as single enzymes. Even though LysT had not shown any significant antimicrobial activity on the agar plate or in suspension, it was active lysin in the zymogram. Perhaps as a VAL, LysT is normally present as a part of a complex or a whole phage, and it is not acting as an antimicrobial lysin. In denaturing SDS-gel, LysT was dissociated from the other phage proteins, and as a free lysin, it could degrade the cell wall of substrate cells. It is not surprising that LysT was only active against LM0230, as its C-terminus is identical to LysP, which also targets LM0230 cells. Therefore, even though VALs may not need a cell wall binding domain for cell targeting and recognition, the high specificity of LysP and LysT indicates that they must have a specific binding region for selecting the target cells. In the zymogram gel, a few other bands were also visible, indicating the presence of autolysins in the concentrated CFS samples. These autolysins could not still be the reason for the antimicrobial action observed in other experiments, as in that case, we should see inhibition zones on agar plates (Figure 3) from all the knock-out mutants. In addition, the zymogram showed that the three phage lysins were able to break the cells without the help of autolysins. However, only LysL gave a band close to its calculated molecular weight of 42.8 kDa, whereas the 26 kDa proteins LysP and LysT gave bigger (approximately 40 kDa) bands than their calculated size. This phenomenon, known as “gel shifting”, is not rare. There can be many reasons why these proteins migrate slower than their expected molecular weight in gel, for instance, amino acid composition, and hydrophobicity. In fact, it has been reported that even 26,8% of proteins migrate at higher than their calculated positions in SDS-PAGE (Shirai et al., 2008). A well-known example of a *Lactococcus* peptidoglycan hydrolase migrating slower than expected in the gel is the 45-kDa protein Usp45, which in SDS-PAGE appears at 60 kDa (van Asseldonk et al., 1990; Hernandez-Valdes et al., 2020). Another atypical phenomenon in the zymogram was LysT giving two bands of around 40 kDa. These two forms of LysT could be a consequence of proteolytic cleavage, or degradation during protein dissociation from the phage, or the two bands may represent different isomeric forms resulting from incomplete reduction of a possible disulfide bridge prior to gel run. Whatever the case may be, the zymographic analysis showed that LysL, LysP, and LysT are able to lyse *Lactococcus* cells as individual proteins, and not (only) as a part of a phage or protein complexes. In addition, the zymogram confirmed the results from other experiments about the peculiar phenomenon of the LAC460 resistance to its own phage lysins. This raises an interesting question of why or how a bacterium carries prophage lysins, which do not target its own cell wall. The further question could be, whether this actually is a common phenomenon, but ignored for some reason.

As shown in Figure 7, the lytic activity in LAC460 supernatant against the *Lactococcus* indicator strains LM0230 and MG1614 started after 4 h and reached its maximum at 7 h after inoculation. After 7 h, lysin activity did not significantly increase. Hence, the lysins seemed to be secreted in late logarithmic and early stationary growth phases. The timing of the secretion is similar to other lytic bacteriocins, e.g., enterolysin A from *E. faecalis*, which was also shown to be secreted in the late logarithmic and early stationary phases (Nilsen et al., 2003). Enterolysin A carries a clear signal peptide, whereas LysL and LysP do not, and thus it is not known how these lysins are secreted. However, the lack of signal peptides in extracellular proteins is a well-known phenomenon. Bacteria have many extracellular or cell surface proteins, for instance, so-called moonlighting proteins, which secretion mechanisms are unknown (Jeffery, 2019). Regardless of the secretion mechanism, our results indicate that the lysins were not released as a result of natural cell death occurring in the stationary/death phase, as in that case, the lytic activity would have increased throughout those phases.

To sum up, prophage lysins behaving like bacteriocins were found to be secreted by the *L. lactis* idli isolate LAC460. Consequently, we propose a new type of bacteriocin into the lytic subclass of the class III bacteriocins: phage lysin-like bacteriocins. As LAC460 lyses other *Lactococcus* strains, it could potentially be useful as an adjunct starter in cheese making to accelerate ripening by releasing enzymes from starter lactococci.

## Data availability statement

The datasets presented in this study can be found in online repositories. The names of the repository/repositories and accession number(s) can be found below: https://www.ncbi.nlm.nih.gov/genbank/, CP059048.

## Author contributions

TT and PS conceptualized the study. TT designed the experiments. TT, SM, and SA carried out the laboratory experiments. TT and XW supervised lab work and performed data visualization. TT and SM wrote the manuscript draft. All authors revised the manuscript and approved the submitted version.
