# Supplementary material for: Wild-type Lactococcus lactis producing bacteriocin-like prophage lysins
Source: Front Microbiol. 2023 Jul 14;14:1219723. doi: 10.3389/fmicb.2023.1219723 (PMC10377672; doi:10.3389/fmicb.2023.1219723)
Supplement: Supplementary file 1 [file Data_Sheet_1.DOCX]

## SUPPLEMENTARY DATA SHEET 1 Amino acid sequences of the three prophage lysins present in *Lactococcus lactis* LAC460 culture supernatant.

LysL, lysozyme + peptidase M23, Locus tag H0A38_RS02765

MKIGTNGLNLIKQFEGCRLTAYNIDDGKITIGWGHAEPVGQTNLVAGVTTWTQAQADNQLTADLVDFENAVNNYFTRSFNQNQFDALVAFAYNLGGGVFANYNWSKTASDSWICSEMILYVNKGTQYEEGLTRRRKAEIDLYNGSSGGSGVSSWTWPFTKPYTGVIYLDGQQFGNTSVKRGRGYFHDGFDFDASVYGPDIFALSDGEVIYTGVMGDGLGSVIVLSIPPYQVMYQEFSQSTSDIFVSVGQKVTRGQRIGRLNGGTHLHLGITQKNWRTALSSWDVDDGSWLNPINVIQDQMSKIIYPKEGEDNMIYAYSADGQPATFLFDGTTTIVFAGANAQPAYNHYVGTYKQIVGKELPNQHKTAQQHALWIQQYPLKYINFK

LysP, peptidase M23, Locus tag H0A38_RS07975

MANDWGWPFSGGYKGYEEGQQFGMTTYDRTGHGDYFHDGFDFGSAKYPGSNIAAVHAGTVVYAGMAPAGYGALGTVVVTKDSSGYYVVYQEFGTSTSNINVSVGQSVSLGQVIGTRNTSHLHLGITKKEWLLAQSSAFKDDGTWLNPINIIQNETGTDNPGPETEGEDEMIKFNVVSGGSKGTAGFLYNGRCIVGGGTGDDNLIYNKLNLMEKTGKIKPIHDDVSGDEYHAMINKFPSFTNGK

LysT, tail-type lysozyme, Locus tag H0A38_RS08355

MGQQETAKQIWDYLTSRGWTQQSVSALLGNMQSESGIIADRWENDNVGNMSGGYGLVQWTPASKYIDWAKSNGLVYQDVISQCKRLEWEVANSQQFYNPNMTFAQFTKSTQTPEDLANIFIKYYERPLNPDQPARAIQARYWYNLFYKNDNNDNKGEDEMIKFNVVSGGSKGTAGFLYNGRCIVGGGTGDDNLIYNKLNLMEKTGKIKPIHDDVSGDEYHAMINKFPSFTNGK
